# Endothelial Progenitors as Tools to Study Vascular Disease

**DOI:** 10.1155/2012/346735

**Published:** 2012-03-15

**Authors:** Reinhold J. Medina, Christina L. O'Neill, T. Michelle O'Doherty, Sarah E. J. Wilson, Alan W. Stitt

**Affiliations:** Centre for Vision and Vascular Science, School of Medicine, Dentistry and Biomedical Science, Queen's University Belfast, Royal Victoria Hospital, Belfast BT12 6BA, UK

## Abstract

Endothelial progenitor cells (EPCs) have great clinical value because they can be used as diagnostic biomarkers and as a cellular therapy for promoting vascular repair of ischaemic tissues. However, EPCs also have an additional research value in vascular disease modelling to interrogate human disease mechanisms. The term EPC is used to describe a diverse variety of cells, and we have identified a specific EPC subtype called outgrowth endothelial cell (OEC) as the best candidate for vascular disease modelling because of its high-proliferative potential and unambiguous endothelial commitment. OECs are isolated from human blood and can be exposed to pathologic conditions (forward approach) or be isolated from patients (reverse approach) in order to study vascular human disease. The use of OECs for modelling vascular disease will contribute greatly to improving our understanding of endothelial pathogenesis, which will potentially lead to the discovery of novel therapeutic strategies for vascular diseases.

## 1. Introduction

There is growing interest in endothelial progenitor cells (EPCs) because of their relevant diagnostic and therapeutic clinical applications. The association of EPCs with cardiovascular events [1] and cancer progression [2] demonstrates that EPCs have potential as both diagnostic and prognostic biomarkers. Furthermore, there are many preclinical and clinical trials that have reported benefits for a cell therapy based on delivering EPCs to ischaemic tissues such as heart [3], brain [4], retina [5], and limbs [6]. In the case of ischaemic heart disease and ischaemic limbs, despite conflicting data, meta-analysis indicated that an EPC-based cytotherapy is feasible, safe, and beneficial [7, 8]. This paper will not further discuss the diagnostic and therapeutic value of EPCs, but will focus on a lesser-known application for EPCs, that is, their potential for modelling human disease pathogenesis. Creating cellular models of human disease is an important research area where EPCs can be readily used and allows for the study of cellular and molecular mechanisms of vascular disease in a “Petri-dish”. Here, we will discuss methodology for EPC isolation and different cell subtypes and also present strategies to use EPCs as valuable tools to model vascular disease.

## 2. EPCs for Disease Modelling

Study of human disease using *in vitro*-based models usually requires large quantities of cells. This is why classically immortalised cell lines had to be established for this purpose. However, these cell lines lack a number of tumour suppressor genes or overexpress oncogenes, which is a major drawback when assessing cellular proliferation and survival. Therefore, recent interest has drifted to the usage of human embryonic stem (hES) and induced pluripotent stem (iPS) cells [9, 10], as they can theoretically be converted into any somatic cell type. We believe that EPCs, as a specific type of adult vascular stem cell [11], have great potential for modelling human disease. EPCs are easily isolated from peripheral and umbilical cord blood, they are highly proliferative, possess a stable and diploid karyotype, represent a very homogeneous cell population that is endothelial lineage-committed, and are amenable to *in vitro* manipulation and genetic modification. In addition, diseases associated with epigenetic changes to cell function can be consistently studied through EPCs, as there is no reprogramming process required, which removes methylation or acetylation events, as is the case for iPS cells.

## 3. Isolation of EPCs

EPCs are isolated using two main methodologies: (a) cell sorting technology using different cell surface markers or (b) *in vitro* cell culture of the blood mononuclear cell fraction using specific substrates and media.

EPC cell sorting is dependent on the type and number of markers used. However, since there is no agreed consensus regarding the most appropriate combination of EPC-linked markers [12], different research teams have been sorting different cells using a diverse array of markers. Therefore, although sorted cells are all named EPCs, they actually represent distinct cell types, and this is demonstrated by the lack of consistency in reported studies using “EPCs” in various *in vitro *and animal model-based systems.

An alternative approach for isolating EPCs is cell culture. This is based on differential adhesion to specific substrates and the subsequent growth potential of isolated cells in culture. Using this methodology, two distinct types of EPCs have been identified [13, 14]. Early EPCs that appear within one week in culture are spindle-shaped cells that exhibit some endothelial properties *in vitro*, such as AcLDL uptake, Isolectin binding, and appearance of VEGFR2/CD31 on the cell surface. Despite these endothelial characteristics, these cells retain their haematopoietic nature, as demonstrated by high expression of CD14 and CD45. In fact, we have recently shown that early EPCs represent M2 alternative-activated macrophages and proposed their renaming as myeloid angiogenic cells (MACs) [15]. Other names for this cell type commonly found in the literature are circulating angiogenic cells, haematopoietic EPCs, proangiogenic monocytes, and vascular accessory cells [16].

The other EPC subtype is known as outgrowth endothelial cells (OECs) [17]. OECs appear within four weeks in culture as a cobblestone-shaped cell monolayer, exhibiting great proliferative potential and an unambiguous commitment to the endothelial lineage [18, 19]. Many studies have clearly described the OEC immunophenotype as being highly positive for the endothelial markers VE-cadherin, vWF, CD31, CD36, CD105, CD146, VEGFR2, and Tie2; negative for haematopoietic markers CD45 and CD14; and exhibit some expression of progenitor cell markers CD34, CD117, and CD133 [5, 17, 20]. OECs are also known as endothelial colony-forming cells (ECFCs), late EPCs, and nonhaematopoietic EPCs. OECs are different from circulating mature endothelial cells due to the fact that they have a higher proliferative potential, shorter doubling time, and single-cell cloning capacity in contrast to mature endothelial cells that have limited proliferative potential [13, 17, 21]. Additionally, OECs retain properties of immature cells, such as greater responsiveness/sensitivity to VEGF, FGF-2, and PlGF [21], and continued expression of progenitor cell markers CD34, CD133, and CD117 [5].

OECs have been shown to possess *de novo* tubulogenic capacity *in vitro *by forming three-dimensional tubular structures where cells interact with each other through the junction protein VE-cadherin and form a distinct vessel-like lumen [22]. This *de novo* blood vessel formation is also demonstrated *in vivo* where human OECs are transplanted subcutaneously in a collagen-fibronectin matrix into immunodeficient mice and efficiently form perfused chimeric blood vessels [18, 23, 24]. Using rhesus monkey-derived OECs in this mouse experimental system, it was recently shown that there was a decreased potential to form functional capillaries with chronological age [25]. Most importantly, it has been demonstrated that OECs directly incorporate into damaged ischaemic vasculature *in vivo* as reported using different animal models such as the murine hind limb ischaemia [13], rabbit carotid artery injury [26], the porcine myocardial infarction [27], and murine retinal ischaemia [5].

For the specific purpose of vascular disease modelling, OECs should be the preferred EPC subtype to use, as they are currently the only EPCs with both great proliferative potential and unequivocal endothelial phenotype.

## 4. Approaches for Disease Modelling with OECs

**Figure 1:**
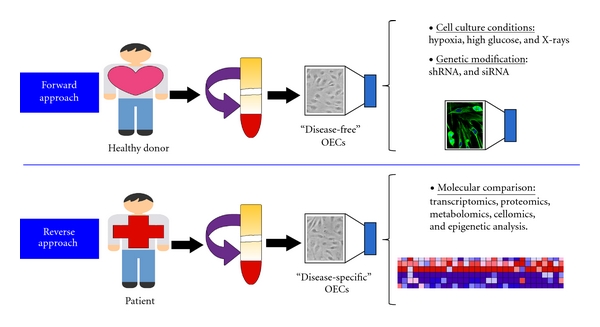
Strategies for the use of EPCs in vascular disease modelling. A specific EPC cell subtype called OEC can be isolated from human peripheral blood of both healthy donors and patients. In the forward approach, “disease-free” OECs are exposed to disease-relevant conditions or genetic modifications, while in the reverse approach “disease-specific” OECs are studied in comparison to “disease-free” OECs.

In disease modelling, the classical “reverse” and “forward” approaches used for hESCs are fully applicable to OECs (Figure 1). The “reverse” approach is based on studying OECs isolated from patients, so that “disease-specific” cells are derived and compared to “disease-free” cells. This approach is very useful as it provides a meaningful insight into physiopathology although it has two drawbacks. First, isolating OECs from certain patient groups may be problematic. For example, it is well known that diabetic patients have a lower number of circulating EPCs and when they are isolated, these cells show dysfunctional responses [28, 29]. The second drawback is that isolated OECs from patients are “already diseased”, and as the “reverse” approach is fundamentally retrospective, it may not be possible to accurately model early stages of a pathogenic process.

The “forward” approach consists of studying “disease-free” OECs that are exposed to defined disease-relevant conditions, which can be as simple as environmental changes (hypoxia, high glucose, and radiation) to more complex genetic modifications by knocking down disease-related genes. This approach is prospective and allows the study of disease pathogenesis from early stages; however, there are some technical challenges. Trying to mimic the pathologic environment can prove very complicated as the *in vivo* milieu usually comprises a diverse variety of factors combined together. Reproducing the *in vivo* environment *in vitro* requires multicell type culture systems. Another difficulty appears when the disease of interest is non-cell autonomous and therefore is directly dependent on different cell-cell interactions, and more than one cell type is needed for disease development and progression. A strategy that could easily address this latter issue of multiple cell types is the adjuvant use of iPS cell methodologies [30]. Generation of iPS cells and OECs from the same donor can provide the means to study OECs in various cell culture settings, including co-cultures with iPS cells or any other iPS cell-derived somatic cell type. This has the advantage that all the different cell types studied alongside OECs will have the same donor which is ideal to avoid possible immunological responses arising from allogeneic transplantation.

While OECs can be studied directly, they can also indirectly facilitate the study of other supportive cells that can modulate vasculogenic activity *in vitro* or *in vivo*. As with fully differentiated endothelial cells, angiogenic activity in OECs can be directed by cytokines released from proximal myeloid cells, mesenchymal stem cells (MSCs) [24, 31], mesenchymal stromal cells, fibroblasts [23], adipose stromal cells [32], pericyte progenitors [33], astrocytes, neurons, and MACs [15, 34]. Interestingly, a mechanism involving the formation of nanotubes for the transport of organelles such as mitochondria and lysosomes has also been reported [35–37]. Delivery of miRNAs within microvesicles and exosomes represent another way cells can communicate with OECs [38, 39].

Despite these technical challenges, utilising OECs to generate cellular models of disease is an attractive methodology that is already being used and optimised. We anticipate that in the field of vascular biology, researchers will favour the use of EPCs/OECs for disease modelling.

## 5. OECs Used As Disease Cellular Models

OECs derived from patients with chronic myeloproliferative disorders (CMD) [40] indicated that this disease targets mainly the haematopoietic system, as the BCR-ABL rearrangement or JAK2-V617F mutation were not present in OECs. This finding highlighted that OECs are not the adult “haemangioblast”, but represent adult stem cells fully committed to the endothelial lineage.

OECs from patients with hereditary haemorrhagic telangiectasia (HHT) [41] revealed abnormalities compatible with vascular lesions, such as decreased endoglin expression, impaired TGF-*β* signalling, disorganised cytoskeleton, and failure to form cord-like structures. These findings described a molecular mechanism to explain small-vessel fragility and frequent bleeding in these patients.

To elucidate the role of EPCs in the pathobiology of pulmonary arterial hypertension (PAH), OECs were isolated from peripheral blood of PAH patients with mutations in the gene-encoding bone morphogenetic protein receptor type II (BMPRII) and control subjects. OECs from PAH patients with BMPRII mutations were hyperproliferative when compared to controls. Furthermore, the matrigel angiogenesis assay demonstrated that *in vitro* tube formation was also significantly impaired in OEC isolated from PAH patients [42].

Von Willebrand disease (vWD) is frequently associated with angiodysplasia; therefore, the importance of vWF expression was tested in endothelial cells and animal models. vWF-deficient cells showed enhanced angiogenesis *in vitro*, and vWF-deficient mice displayed increase angiogenesis *in vivo*. These results were further confirmed by isolating OECs from patients with vWD [43] which showed increased *in vitro* angiogenesis, proliferation, and migration.

To study the role of the diabetic environment in EPC function, OECs were exposed to high glucose, and umbilical cords of diabetic mothers were used as the EPC source to isolate OECs that had previously experienced diabetic conditions *in vivo *[44]. Results demonstrated that exposure to high glucose *in vitro* or a diabetic environment *in vivo* significantly diminished OEC function such as colony formation, self-renewal capacity, and capillary-like tube formation. This study provided potential mechanistic insights into the long-term cardiovascular complications observed in newborns of diabetic pregnancies.

## 6. Concluding Thoughts

OECs are a specific EPC sub-type that is starting to be used for the study of vascular pathology. We encourage researchers in the field of vascular biology to apply their different *in vitro* and *in vivo* models of angiogenesis to OECs. Combination of forward and reverse approaches for human disease modelling with OECs is an effective system for the study of vascular disease pathogenesis. As with any new technology, we foresee some technical challenges when establishing disease models at the cellular level; nevertheless, we remain optimistic that utilising OECs for vascular disease modelling will improve our understanding of disease that subsequently leads to the development of novel therapies.
